# A new biphasic osteoinductive calcium composite material with a negative Zeta potential for bone augmentation

**DOI:** 10.1186/1746-160X-5-13

**Published:** 2009-06-13

**Authors:** Ralf Smeets, Andreas Kolk, Marcus Gerressen, Oliver Driemel, Oliver Maciejewski, Benita Hermanns-Sachweh, Dieter Riediger, Jamal M Stein

**Affiliations:** 1 Department of Oral and Maxillofacial Surgery, University Hospital Aachen, Aachen, Germany; 2 Interdisciplinary Center for Clinical Research (IZKF) 'BIOMAT', RWTH Aachen University, Aachen, Germany; 3 Department of Oral and Cranio-Maxillofacial Surgery, Technische Universität, Klinikum rechts der Isar, Munich, Germany; 4 Department of Oral and Maxillofacial Surgery, University of Regensburg, Regensburg, Germany; 5 Department of Pathology, University Hospital Aachen, Aachen, Germany; 6 Department of Operative Dentistry, Periodontology and Preventive Dentistry, University Hospital Aachen, Aachen, Germany

## Abstract

The aim of the present study was to analyze the osteogenic potential of a biphasic calcium composite material (BCC) with a negative surface charge for maxillary sinus floor augmentation. In a 61 year old patient, the BCC material was used in a bilateral sinus floor augmentation procedure. Six months postoperative, a bone sample was taken from the augmented regions before two titanium implants were inserted at each side. We analyzed bone neoformation by histology, bone density by computed tomography, and measured the activity of voltage-activated calcium currents of osteoblasts and surface charge effects. Control orthopantomograms were carried out five months after implant insertion. The BCC was biocompatible and replaced by new mineralized bone after being resorbed completely. The material demonstrated a negative surface charge (negative Zeta potential) which was found to be favorable for bone regeneration and osseointegration of dental implants.

## Background

To place an implant in the posterior upper jaw often requires augmentation procedures in order to increase the vertical dimension and obtain sufficient anchorage of the implant in the alveolar bone that bears the implant. A common method to increase the amount of bone that receives the implant at the lower portion of the sinus floor is maxillary sinus floor augmentation with bone grafts [1,2]. A variety of bone grafts and bone replacement materials have been recently used for this procedure. Autologous bone has been considered to be the gold standard [3,4]. However, a second surgical site is needed in order to harvest the bone; the size of the graft is often limited, and donor site morbidity frequently represents a problem. For this reason, bone replacement biomaterials such as demineralized freeze-dried bone allografts and xenografts have been recently used for sinus augmentation with good clinical results and various authors recently reported on the resorption and remodeling of the materials [5,6]. Although it seems to be statistically negligible, there is still a risk to transmit diseases by using xenografts. Therefore, much effort is made to create alternative materials. The development of more effective alloplastic materials may be a useful alternative way.

**Figure 1:**
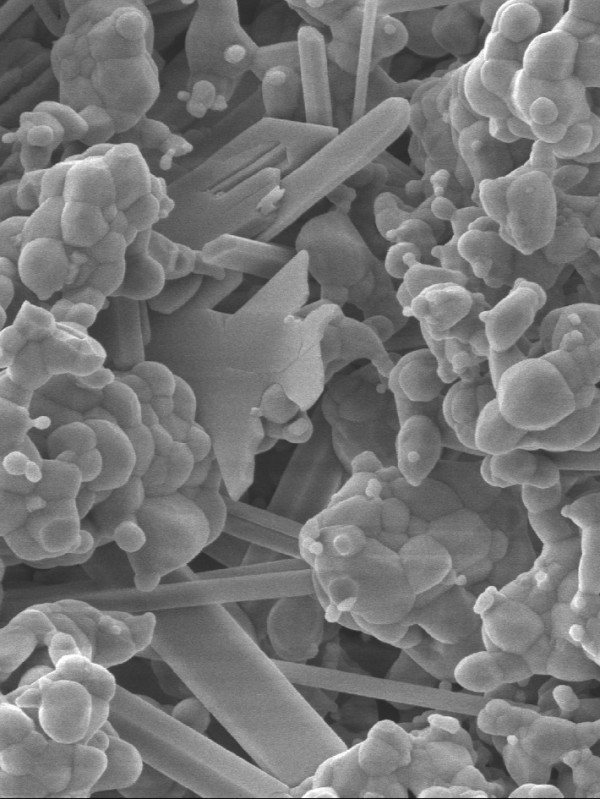
**Scanning electron micrograph image of the biphasic calcium composit graft material shows inherent microporosity and fully interlocked microstructure**. This microstructure provides the mechanical stability. The two separate crystalline components are clearly identified.

Among alloplastic biomaterials, hydroxyapatite, tricalcium phosphate (TCP) (α- and β-TCP) and calcium sulfate have been used as bone substitutes. In comparison to some of the hydroxyapatites, it has recently been shown that β-TCP and calcium sulfate were resorbable [7-9] and that they have osteoconductive properties [10,11]. Previous studies indicated that sinus floor augmentation using β-TCP lead to remodeling and formation of new bone. However, the volume of bone formation was significantly reduced in comparison to autologous bone grafts [7,12]. First results of the clinical application of a new biphasic calcium composite (BCC) bone graft material (Fortoss Vital, Biocomposites Ltd., Keele Staffordshire, England) in reconstructive periodontal and periimplant surgery were promising in terms of the osteoconductive and osteogenetic potential of the BCC material [13,14]. BCC consists of a porous β-tricalcium phosphate and a bacteriostatic calcium sulfate phase (Fig. 1). Due to a modified surface activity and ion loading, its osteoconductive behavior seems to be superior if compared to conventional calcium phosphates. Zeta Potential Control (ZPC™; Biocomposites Ltd., Keele Staffordshire, England) is the proprietary process to produce a bioactive bone graft material with a controlled negative surface charge. When placed into healthy bleeding bone, this negative charged surface accelerates the bone growth cascade [15,16]. Several factors determine the body's initial response to an implanted bone graft material (the host response). Cells will not interact directly with the surface of the implanted material, either in-vitro or in-vivo [11], and the process of cell interaction with an implant material is highly dynamic.

Surface charge is one of these factors, as bone acquires a surface charge when brought into contact with an aqueous environment. The separation in charge between the solid phase of bone and the body's extracellular matrix creates a potential difference between the solid and the liquid called the Zeta potential, which influences the type and nature of proteins and cells harnessed by the surface. The magnitude of the negative polarity of the zeta potentials of the BCC increases with increasing sintering temperature [17]. Various researchers have already reported that negatively charged surfaces have a positive effect on the heterogeneous nucleation of alloplastic materials in a supersaturated simulated body fluid solution, whereas this nucleation is inhibited on positively charged surfaces [18]. This phenomenon is believed to be caused by the electrostatic accumulation of Ca^2+ ^ions near the negatively charged surfaces, which seems to trigger the initial nucleation.

**Figure 2:**
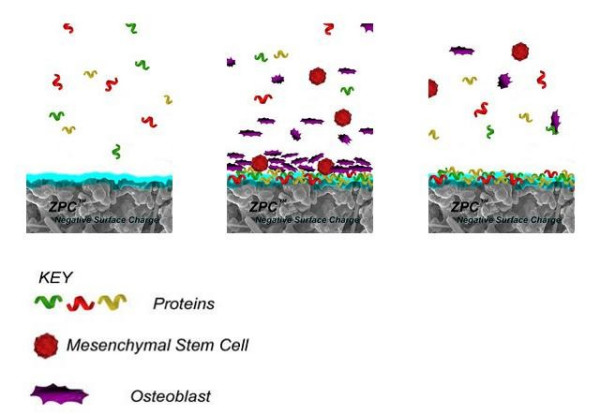
**ZPC™ Graft surface activation**. Step 1. Negative surface charge interacts with the extracellular matrix. Step 2. Protein adsorption at the charged surface. Step 3. Cell attachment and proliferation.

The ZPC™ process enables the manufacturing of a synthetic bone graft with a controlled negative surface charge (Fig. 2). The analysis of this biomaterial concerning the suitability for sinus floor augmentation has not been studied yet.

The purpose of our study is to analyze and demonstrate the osteogenic, histologic and radiographic qualities of the BCC bone graft fabricated through the Zeta potential process, which enables production of a bioactive bone graft material with a controlled negative surface charge and which, when placed in apposition to healthy bleeding bone, accelerates the bone growth cascade [15,16].

## Methods

### Clinical background and surgical procedure

**Figure 3:**
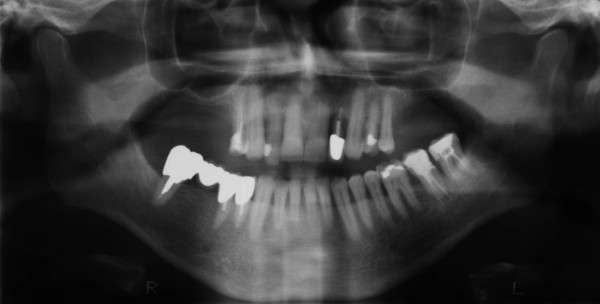
**Pre-operative panoramic radiograph**.

**Figure 4:**
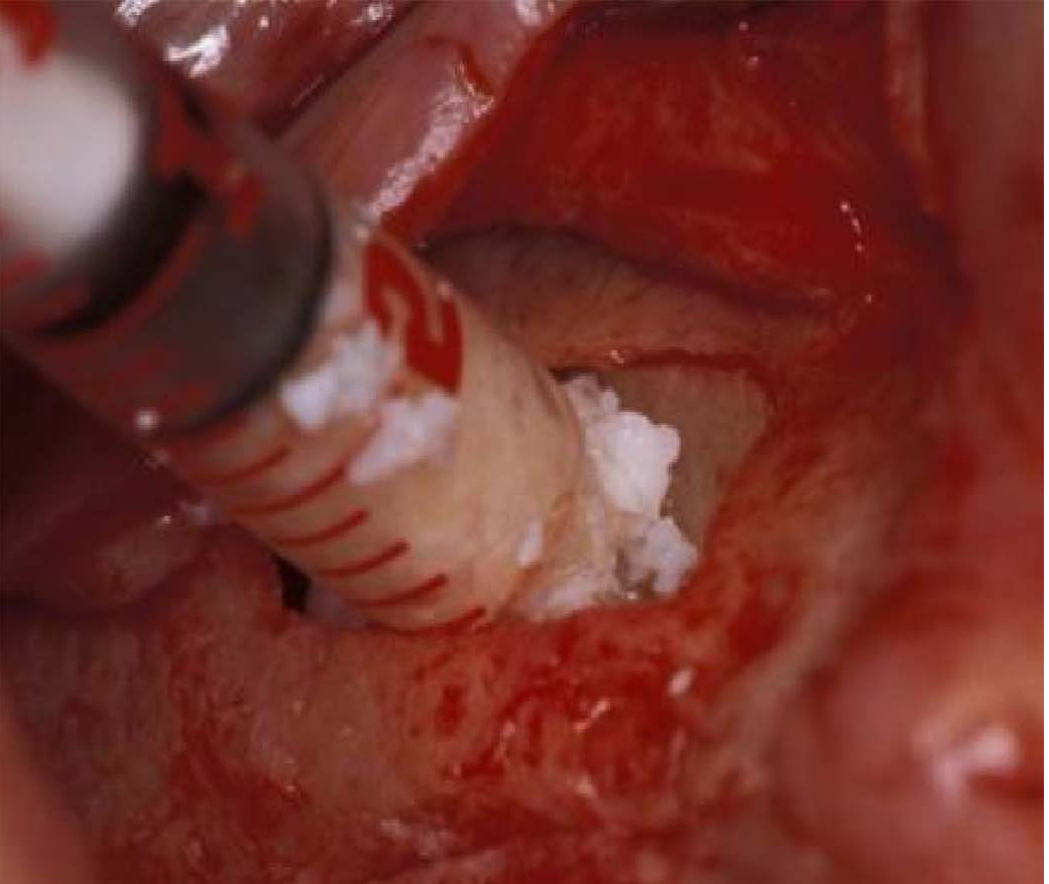
**Sinus floor augmentation with the biphasic calcium composite material**.

Due to generalized horizontal bone loss and chronic periodontitis in the upper and lower jaw (Fig. 3), standardized maxillary sinus floor augmentation was performed prior to the placement of dental implants in a 61 year old male patient. The molars of the upper jaw and the second premolar in the first quadrant were missing. Due to a bone thickness of the sinus floor of less than 4 mm, a two-stage surgery was indicated. Both the right and left sinus were augmented according to the method of Boyne and James [1]. In brief, a full thickness mucoperiosteal flap was detached; a rectangular window was reamed into the lateral sinus wall and infractured. The internal mucosa that covers the maxillary sinus was carefully elevated and displaced inwards (towards medial) together with the bone window. The space beyond the prepared membrane and bone window was filled with the BCC bone graft (Fig. 4). The material was compacted and the mucoperiosteal flap was readapted and sutured. The postoperative period was uneventful. Six months after the augmentation of both sinuses, two 11 × 4 mm implants (Osseotite Certain; 3i Implant Innovations; West Palm Beach, FL, USA) were placed on each side in the augmented regions corresponding to the first right upper molar, second right upper premolar and first and second left upper molars. Prior to the insertion of the implants, dental computed tomography (CT; Twin Elsent, Maconi Philips, Best, Netherlands) was made in order to evaluate the dimension and density of the augmented sinus floors.

### Analysis of voltage-activated calcium currents

Patch clamp physiological techniques were used to characterize the voltage-activated calcium currents expressed in the plasma membrane of osteoblastic cells which are influenced by the surface loading of the bone substitute. Preparations enriched with osteoblasts were isolated by collagenase digestions of newly formed bone and cultured under different conditions which affected cell proliferation. The Zeta potential was measured by using the Zetasizer 3000 device (Malvern, Herrenberg, Germany). Ethanol, isopropanol and methanol were used as liquid phases in reagent grade. Measurements were performed in saturated calcium phosphate solution, in 0.05 mol/l sodium phosphate solutions and in deionized water. The potential was determined six times; the mean values and standard deviations were calculated. The initial setting time of the cements was measured according to the Gilmore needle test in a humidity chamber at 37°C and a humidity of >90% [19].

### Sample preparation

During implant placement, a bone biopsy was taken from the augmented area in the first quadrant using a trephine bur. In order to focus on the augmented region, only the cranial bone portion of the bone cavity for the 4 × 11 mm implant was harvested resulting in a 4 × 6 mm sample which was available for histologic evaluation. The specimen was immediately rinsed in saline, fixed in 4% formalin in phosphate buffer for 24 hours at room temperature and demineralized by using EDTA solution. The samples were cut (1–2 μm thickness) and stained in hematoxylin-eosin and Ladewig stain. Microscopic evaluation was carried out on light microscopy in the magnifications 25×, 100× and 400×.

## Results and discussion

### Zeta potential

The BCC material demonstrated a negative Zeta potential in organic media (-5.2 ± 1.3 mV in ethanol and -3.4 ± 1.4 mV in isopropanol), as well as in aqueous antibiotic solution [-20.4 ± 2.1 mV in gentamicine and -24.6 ± 1.8 mV in amoxicillin]. For comparison, it demonstrated -19.6 ± 1.1 mV in water. The particle surfaces only showed a slight electrostatic charge in the solvents. The mean particle size was 1.9 to 2.1 (mm) depending upon the organic suspension medium. According to the value of the Zeta potential, the minor particle size correlated with an increasing electrostatic charge of the particle surface.

### Histology

**Figure 5:**
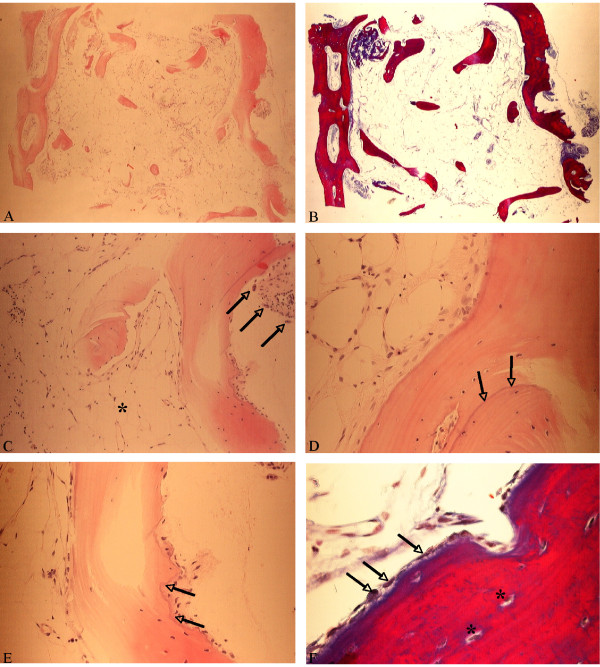
**Histologic sections of the sample A, Overview of the sample in H&E, B, and in Ladewig staining (both magnification × 25)**. C, Signs of bone remodeling with presence of osteoclasts (arrows) in higher magnification, surrounding bone marrow without fibrosing and fatty degeneration(*), H&E, magnification ×40. D, Sporadic cement lines indicating new bone formation (arrows), H&E, magnification ×100. E, A small osteoid line borders the bone trabecules, H&E, magnification ×100 F, In Ladewig stain this line appears in blue color and is margined by a layer of osteoblasts (arrows), magnification ×400.

During the procedure of implant site preparation in the first quadrant, a tissue specimen of 4 × 6 mm was taken and stored for further analysis in 4% formalin in phosphate buffer. The sections of the biopsy showed a portion of the bone tissue, characterized by marginally compact trabecular bone with regular lamellar and woven structure as well as a fatty non-fibrosing bone marrow (Fig. 5). No BCC particles could be identified. However, sporadic osteoclasts indicated a preceding resorption activity (Fig. 5c) and little cementum lines showed the formation of new mineralized bone (Fig. 5). A thin strand of osteoid, stained in blue color in Ladewig stain (Fig. 5), represented the margins of the bony trabecules and was surrounded by osteoblasts. Singular lymphocytes were also visible in the bone marrow. There were no signs of acute inflammatory or foreign body reactions.

### Dental computed tomography

**Figure 6:**
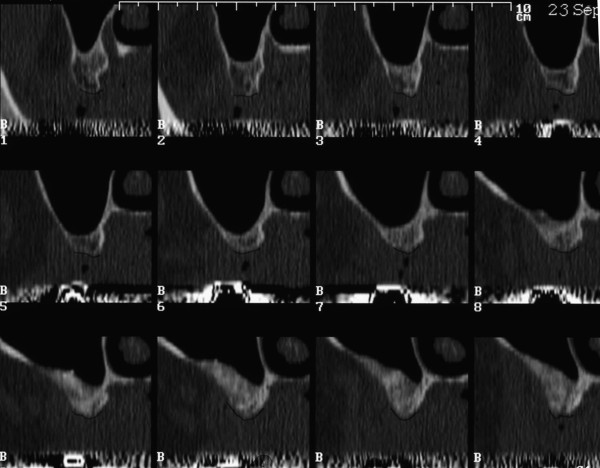
**Dental-CT: Layers B1 – B12 = regio 18 – 14; Distance between the layers: 2.5 mm (1:1)**.

The bone quality of the augmented sinus floor was evaluated in a dental CT of the upper jaw prior to implant placement (Fig. 6). Radiological signs of marginal osseointegration of the graft could be shown. Bone density was assessed by using the Hounsfield classification. The measurements corresponded to a bone quality of Q3 to Q2. There were no radiological signs of inflammation visible. In the region of the roots of the upper jaw, a regular bone structure was found. Also, sinus maxillaris was free of inflammation and was normally ventilated.

### Panoramic radiograph

**Figure 7:**
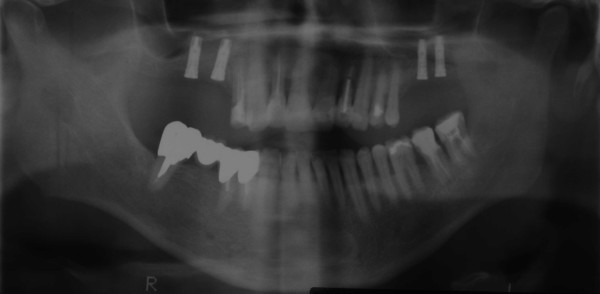
**Panoramic radiograph five months after implant insertion**.

A postoperative panoramic radiograph (orthopantomogram) was made five months after the insertion of the implants prior to crown restoration on the implants (Fig. 7). The radiograph shows gain of sufficient bone volume in the sinus floor area of both sides surrounding the osseointegrated implants. The augmented sinus floors were characterized by homogenous bone with a regular trabecular structure and similar density compared to the residual bone. No signs of inflammation were evident.

The surface potential of bone substitutes in direct contact to bone is of great interest in the recent time. However, until now the exact system of the implant to bone bond can not completely be explained. Surface functional groups such as carboxyl and hydroxyl ions determine the surface charge, the degree to which such surface groups alter the resulting bone substitute to osteoblast or precursor cells or bone is not fully understood [20]. To our knowledge this is the first report of biphasic calcium composite material for sinus floor augmentation. The clinical, radiographic, histological and patch clamp results of our study suggest that this biomaterial might be a more effective bone substitute for maxillary sinus augmentation than other alloplastic bone substitutes. The histological analysis six months after sinus augmentation showed osteoblasts and evidence of new bone formation in the augmented regions. Moreover, enhanced presence of osteoclasts revealed the tendency of ongoing accelerated resorption processes. Interestingly, there were no BCC particles visible anymore, suggesting a fast and complete remodeling of the material.

There are only limited data available on the dynamics of resorption of bone replacement materials after maxillary sinus floor augmentation. For bovine hydroxyapatite, a slow resorption is known to occur after maxillary sinus floor augmentation. Although in animal models [21,22] and in humans [6] an increase of new bone formation in an osteoconductive manner has been shown [23], in most studies available bovine hydroxyapatite particles were not or not fully absorbed after a period of six months [24], twelve months [6,21,25] or even later [25]. Similar findings were made with the use of conventional tricalcium phosphates. However, data suggest a faster resorption rate with these materials [23,26,27]. In a case control study, Eggli *et al. *showed a TCP resorption of 85% compared to 5.4% for hydroxyapatite six months after implantation in cancellous bone of rabbits [28]. In a comparative histomorphometric analysis, Artzi *et al. *reported a better resorption rate of TCP than bovine hydroxyapatite [29]. However, a complete resorption of calcium phosphate particles with simultaneous osteoconductive bone formation six months after sinus augmentation as shown in the present study with the BCC has not been reported yet. The reason for this observation might be based on special surface properties of this biomaterial. Both components of the BCC, the porous calcium phosphate and implant grade calcium sulfate, biodegrade within weeks to months. Biodegradation of calcium sulfate leads to a developing macroporosity. To replace the initial microporosity in between calcium phosphate particles, cells and nutrient fluids draw in [30]. Calcium phosphate particles are then able to interact with osteogenic cells. In this interaction, the ZPC™ principle plays an important role [13].

Based on this unique concept, the surface of the material will be charged negatively in an aqueous environment. A number of studies have shown that a material that has an electronegative surface charge (negative Zeta potential) is more accessible for the attachment and proliferation of osteoblasts than surfaces with no or even positive electric charge [31-34]. The Zeta potential as one of the surface properties is strongly influenced by the reactivity of the calcium phosphate particles. It plays an important role due to the manufacturing and application process of the fine grain powder mixtures of BCC. Suspension media leading to a high surface charge of the Zeta potential and particle sizes of this material ground in several organic media particles lead to electrostatic stabilization of the particle surface, minimized agglomerization resulting in the splitting of the particles instead of agglomerates. As a reaction, small rapidly diffusing positively charged proteins initially attach to the graft material. These are then replaced by larger, positively charged proteins with a strong affinity to the surface that show chemotactic and adhesive properties. Through this mechanism of adsorption of positively charged proteins at the surface, the material is accessible for a rapid attachment of osteoblasts [35] as the negatively charged species (mesenchymal cells and osteoblasts) are drawn into intimate contact [36]. Once the osteoblasts have attached, they undergo the process of cellular adhesion which is slower than the adsorption of charged proteins. During cellular adhesion, the osteoblasts accomplish a true biological attachment, enable cell proliferation and benefit bone formation (Fig. 2).

For the BCC material presented in this study, the formation of a negative Zeta potential had recently been demonstrated in vivo and in vitro: Cooper and Hunt evaluated the expression of selected osteogenic markers (alkaline phosphatase, osteocalcin, osteopontin, core binding factor alpha-1 (CBFA1) and collagen type 1) in vitro by reverse transcription-polymerase chain reaction (RT-PCR) in a culture of osteoblasts in contact to different calcium phosphate materials with positive and negative Zeta potential values [37,38]. They demonstrated a strong correlation of a negative Zeta potential with the expression of several osteogenic markers. Other authors recently reported on the significance of relative Zeta potentials of bone and different biomaterials and their influence on protein adsorption [34]. They demonstrated that the adsorption of specific extracellular matrix proteins onto biomaterial surfaces provided sites for an integrin-mediated osteoblast attachment. In the case of BCC with its negatively charged surface, all osteogenic markers were expressed while the conventional pure phase TCP with a positively charged surface only induced the expression of osteopontin and alkaline phosphatase. Moreover, it is well known that rapid resorption of the calcium sulfate matrix alone promotes osteogenic activity due to formation of a calcium phosphate lattice [39,40] promoting osteogenic activity. It mimics the mineral phase of bone and is resorbed at the rate of bone formation [41].

The density of the newly formed bone corresponded to a quality of Q2 to Q3 according to the Hounsfield classification, as verified by determination of the specific Hounsfield units by CT scans. This quality of bone can be compared with the local origin trabecular bone and is even denser than the original bone matrix of the posterior maxillary region. This confirms our histologic findings and the stable osseointegration of the implants, which are surrounded by sufficient bone as seen in the postoperative panoramic radiograph.

The authors are aware of the limitations of the present study, whose data base on the analysis of samples from one patient. Therefore, we regard this report as to indicate a trend. We also cannot draw general conclusions or definitive statements about the possible shrinkage of the grafted volume due to degradation of BCC, which has been observed with other bone replacement materials [42,43]. From a practical point of view, however, we felt that careful but sufficient compaction of the material during sinus augmentation might contribute to minimize a possible shrinkage due to resorption processes. Nevertheless, this study indicates previously unknown properties of a new composition of two materials with a special biodegradation dynamic and surface potential as this could be demonstrated by the patch clamp technique. Nevertheless, the presented experiences are encouraging since the final aim of the maxillary sinus floor augmentation procedure is osseointegration of implants into vital bone.

## Conclusion

We could show that BCC is a promising and effective alloplastic biocompatible bone replacement material with superior osteoconductive properties due to the negative Zeta potential. The negative Zeta potential has biological important effects *in vivo*. BCC seems to be very suitable for maxillary sinus floor augmentation and allows stable osseointegration of dental implants compared to conventional pure phase β-TCP. Case control studies with histomorphometric analysis should confirm the promising preliminary results and might verify further indications.

## Competing interests

The authors declare that they have no competing interests.

## Authors' contributions

RS and JMS conceived the study, participated in the design of the study and coordinated the work. MG contributed to the analysis, interpretation and discussion of the data and performed the proofreading of the manuscript. OM, AK, BH, DR and OD conceived the study, participated in the design of the study, carried out experimental work. All authors read and approved the final manuscript.
